# *Francisella tularensis* human infections in a village of northwest Iran

**DOI:** 10.1186/s12879-021-06004-y

**Published:** 2021-03-31

**Authors:** Saber Esmaeili, Mahdi Rohani, Ahmad Ghasemi, Mohammad Mehdi Gouya, Simin Khayatzadeh, Ahmad Mahmoudi, Hossein Ahangari Cohan, Anders Johansson, Max Maurin, Ehsan Mostafavi

**Affiliations:** 1 National Reference laboratory for Plague, Tularemia and Q fever, Research Centre for Emerging and Reemerging infectious diseases, Pasteur Institute of Iran, Akanlu, Kabudar Ahang, Hamadan Iran; 2 Department of Epidemiology and Biostatistics, Research Centre for Emerging and Reemerging infectious diseases, Pasteur Institute of Iran, Tehran, Iran; 3 Department of Microbiology, Pasteur Institute of Iran, Tehran, Iran; 4 Department of Bacteriology, Faculty of Medical Sciences, Tarbiat Modares University, Tehran, Iran; 5 Center for Communicable Disease Control, Ministry of Health and Medical Education, Tehran, Iran; 6 Department of Communicable Disease Control, Deputy of Health, Tabriz University of Medical Sciences, Tabriz, Iran; 7 Department of Biology, Faculty of Science, Urmia University, Urmia, Iran; 8 Department of Clinical Microbiology and Molecular Infection Medicine Sweden (MIMS), Umeå University, Umeå, Sweden; 9 Universite Grenoble Alpes, CNRS, Grenoble INP, CHU Grenoble Alpes, TIMC-IMAG, 38000 Grenoble, Grenoble, France

**Keywords:** *Francisella tularensis*, Water, Tularemia, Rodent, Chlorination

## Abstract

**Background:**

Recent seroepidemiological studies have suggested that tularemia could be an endemic bacterial zoonosis in Iran.

**Methods:**

From January 2016 to June 2018, disease cases characterized by fever, cervical lymphadenopathy and ocular involvement were reported in Youzband Village of Kaleybar County, in the East Azerbaijan Province, northwestern Iran. Diagnostic tests included *Francisella tularensis* serology (including tube agglutination test and ELISA), PCR, and culture.

**Results:**

Among 11 examined case-patients, the tularemia tube agglutination test was positive in ten and borderline in one. PCR detected the *F. tularensis* *ISFtu2* elements and *fopA* gene in one rodent and a spring water sample from the same geographic area.

**Conclusions:**

Based on the clinical manifestations of the disease suggesting an oropharyngeal form of tularemia, serology results in case patients, and *F. tularensis* detection in the local fauna and aquatic environment, the water supply of the village was the likely source of the tularemia outbreak. Intervention such as dredging and chlorination of the main water storage tank of the village and training of villagers and health care workers in preventive measures and treatment of the illness helped control the infection.

## Introduction

Tularemia is a zoonotic disease caused by the Gram-negative bacterium *Francisella tularensis* [1]. The disease classically manifests by six clinical forms, the ulceroglandular, glandular, oculoglandular, oropharyngeal, respiratory, and typhoidal forms. The ulceroglandular and glandular forms correspond to chronic lymphadenopathy, with or without a skin inoculation lesion, respectively. The oropharyngeal form corresponds to pharyngitis with cervical lymphadenopathy. It is mainly caused by the consumption of contaminated water or food. The oculoglandular form is a conjunctivitis with regional lymphadenopathy, usually transmitted through the touch of eyes with contaminated fingers or by an ocular projection of contaminated water or dust. The respiratory and typhoidal forms are usually more severe systemic diseases [1, 2].

*F. tularensis* has three subspecies, namely *tularensis* (type A), *holarctica* (type B), and *mediasiatica* that are different in terms of reservoirs, life cycle, and geographic distribution. Only type A and type B strains are known causative agents of tularemia in human. Type A strains are reported in North America, and type B is common in Europe and Asia [1]. Two aquatic and terrestrial cycles have been described for both subspecies [3–5]. In the terrestrial cycle, rabbits and hares are hosts and reservoirs of the disease, and ticks play a major role as vectors in the animal reservoir. In the aquatic cycle, which is more common for type B strains, small rodents contaminate the aquatic environment with *F. tularensis*, and tularemia can be transmitted to humans by drinking contaminated water, and in some areas (Sweden, Finland and Russia) through mosquito bites. However, type B strains can also be found in hares, rodents and other animals [3]. In regions with type B tularemia, drinking water from ponds, lakes and rivers and eating contaminated vegetables can transmit the disease [4, 6].

Tularemia is classically restricted to the Northern hemisphere, although it has been recently reported in Australia [7]. It is more common in the USA and Northern Europe (Scandinavia) [1], and disease foci are found in Russia, Kazakhstan, and Turkmenistan [8, 9]. Tularemia has been reported in animals or humans in several countries with common borders with northern Iran, such as Turkmenistan, Kazakhstan, Azerbaijan and Armenia [10]. This disease is highly endemic in Turkey [11], where 1300 human infections have been reported in different regions during the 1988 to 2009 period [6, 12].

The first serosurvey of tularemia in wild mammals and livestock from different regions of Iran, in 1972, showed evidence of *F. tularensis* circulation among sheep and cows in the northwestern part of Iran, but also in a hedgehog in the southeastern part [13]. In 1980, the first human case of tularemia (a glandular form) was reported in Kurdistan province, in western Iran [14]. Since 2011, with the establishment of the national tularemia laboratory in the Pasteur Institute of Iran, extensive studies have been done in this field in Iran. According to conducted serosurveys on Kurdistan province residents during 2011–2012, 14.4% of studied participants had anti-*F. tularensis* antibodies [15]. In 2011, the seroprevalence of tularemia was found to be 6.52% among butchers and slaughterhouse workers of Sistan and Baluchestan province, southeastern Iran [16]. In 2013, a seropositive rodent was found in the northern Sistan and Baluchistan [17]. During another research in Kurdistan province in 2014, seropositive tularemia samples were reported among rodents [18]. In 2014 and 2015, rodents trapped in the Golestan (northern Iran), Khuzestan (southwestern Iran) and Razavi Khorasan (northeastern Iran) provinces, and hares from Khuzestan, and Sistan and Baluchestan provinces (southeastern Iran) were PCR-positive for *F. tularensis* [19]. In 2017, a human case of hare-borne tularemia was diagnosed in Kurdistan province [20].

According to previous studies and current epidemiological data in neighboring countries such as Turkey, an aquatic cycle could be suspected in Iran.

## Materials and methods

### Outbreak site

Youzband is a village of East Azerbaijan Province in the northwest of Iran, located 18 km from Kaleybar County. The village’s population was 719 in 2017. The village is situated at 1600 m elevation above sea level.

### Reported cases

**Fig. 1:**
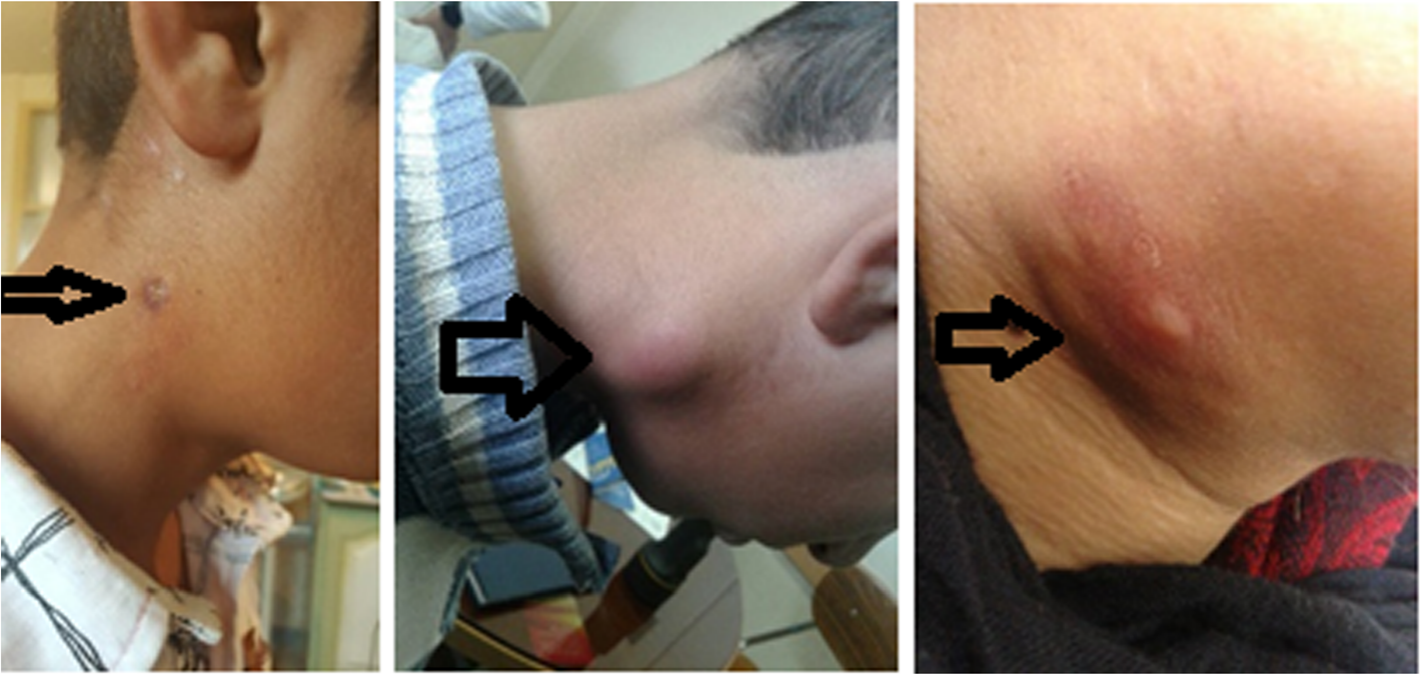
Cervical adenopathy in some tularemia patients

From December 2016, a disease was observed in the village affecting humans from different families and of different ages. Clinical manifestations included fever, fatigue, muscle aches, and the development of swollen lymph nodes, mainly in the neck. Thirteen patients had been suffering from this disease, according to the recorded data at the village health House (Fig. 1).

Patients were referred to the provincial and hospital centers in Kaleybar County for diagnosis, and most of them received antibiotics such as amoxicillin, coamoxiclav, penicillin, ceftazidime, and cefixime. In some cases, surgical lymph node removal was performed. Two patients received anti-tuberculosis treatment despite a negative acid fast-staining of histological preparation of removed tissue samples. Some patients did not receive any treatment as the swollen lymph nodes drained into or out of the body. With regard to clinical manifestations, four pathogens were suspected in the differential diagnosis by physicians, including *Francisella tularensis, Yersinia pseudotuberculosis, Yersinia pestis and Bartonella henselae*.

### Environmental observations and epidemiological evidence

A water-borne disease was strongly suspected during the investigation carried out in the village by the epidemic control team of the Pasteur Institute of Iran. No similar disease was found in surrounding villages. The investigation team found out that the villagers of Youzband village preferably used drinking water from local springs because they disliked the water of the local water supply system having an unpleasant taste and color. Data from registry offices indicated that the chlorination of the village tap water was insufficient 1 year before the disease emergence. In interviews with villagers, an unusual abundance of rodents in the summer of 2016 was frequently mentioned.

Given epidemiological, environmental and clinical findings, the suspicion of a tularemia outbreak became more serious, and it was decided to perform clinical diagnostic sampling of humans and send serum, blood and lymph nodes specimens to the Pasteur Institute of Iran.

### Specimens for microbiological testing

Among humans with a clinical history of suspected tularemia manifestations during the previous 18 months, eleven were still ill and expressed their consent to provide blood samples at the time of the outbreak investigation in June 2018. We collected serum samples from all patients, whole blood samples from six patients, and five samples from village’s water resources, including spring water and tap water. In addition, nine rodents were captured from around the water resources, and rodent serum and spleen tissue samples were collected.

### Demographic and clinical data of patients

A researcher-made survey questionnaire was used to collect information from the patients. The patients’ mean age was 30 years (the youngest was 4, and the oldest was 77). The swollen lymph nodes were retropharyngeal in six patients, parotid in three, and at both locations in two patients. One patient had swollen inguinal lymph nodes. In two patients, there was no swelling of lymph nodes but swelling and redness of the eyes. Physical examination of the patients revealed redness of the oropharyngeal mucosa. The disease was accompanied by cervical and preauricular swollen lymph nodes, usually unilateral. In four patients, lymph node swelling had disappeared, resulting in spontaneous skin drainage with pus suppuration.

Seven patients reported the onset of symptoms in 2016 (including two in December) and two patients in 2017 (in March and October, respectively). Two patients with severe illness required hospitalization. Two patients consumed only spring water, three only tap water, and six drank both spring and tap water. Two patients pointed out that another member of their families had also been affected by the same clinical manifestations (Table 1).

**Table 1:** Demographic, epidemiological and clinical data and test results for 11 patients involved in tularemia outbreak in Youzband Village, 2016–2018

Case No.	Age/Gender	Lymphadenopathy	Wound in lymph node	Ocular lesion	Primary signs date	Clinical sign	Treatment/Duration	Drinking Water	Keeping livestock	History of tick biting	Serum sampling time (at days post onset)	Tube agglutination test	IgM titre U/ml (ELISA)	IgG titre U/ml (ELISA)
**1**	**29/M**	**Pharyngeal**	**N**	**Y**	**Nov. 2016**	**Eye discharge, chills, sore throat, swollen lymph nodes**	**Clindamycin, Amoxicillin/ 1 week**	**Only Spring**	**Y**	**N**	**574**	**1:320**	**≥400**	**235**
**2**	**44/F**	**Pharyngeal**	**Y**^**a**^	**N**	**Dec. 2016**	**Enlarged lymph node, Common Cold sign**	**Cefixime, Metronidazole Hydrocortisone ceftriaxone/** **2 month**	**Only Spring**	**Y**	**N**	**568**	**1:160**	**≥400**	**190**
**3**	**30/F**	**Preauricular**	**N**	**N**	**Nov. 2016**	**Fever, Chill, Sore throat, weakness, enlarged lymph node**	**Anti TB drugs/ ND**	**Tap water & spring**	**N**	**N**	**575**	**1:320**	**≥400**	**170**
**4**	**30/F**	**Pharyngeal**	**N**	**N**	**Nov. 2016**	**Sore Eyes,** **enlarged lymph node**	**Penicillin/ ND**	**Only spring**	**N**	**N**	**574**	**1:320**	**≥400**	**84**
**5**	**29/F**	**Pharyngeal& Preauricular**	**N**	**N**	**Dec. 2016**	**Common cold sign, Anorexia Fever, Chill, sore throat, enlarged lymph node**	**Not treated**	**Only Tap water**	**Y**	**N**	**566**	**1:40**	**260**	**145**
**6**	**4/M**	**Preauricular**	**Y**	**N**	**Dec. 2016**	**Sore throat, enlarged lymph node (Hospitalized)**	**Cephalexin clindamycin clarithromycin ciprofloxacin/ ND**	**Tap water & spring**	**Y**	**N**	**567**	**1:320**	**240**	**260**
**7**	**8/M**	**Inguinal & pharyngeal**	**N**	**N**	**Nov. 2016**	**Sore throat, skin rash on feet (Hospitalized), enlarged lymph node**	**Betamethasone/ ND**	**Tap water & spring**	**Y**	**N**	**574**	**1:160**	**250**	**230**
**8**	**14/M**	**Pharyngeal**	**Y**	**N**	**Dec. 2016**	**Common cold sign, enlarged lymph node**	**Not treated**	**Tap water & spring**	**Y**	**Y**	**569**	**1:320**	**95**	**≥300**
**9**	**31/F**	**Pharyngeal**	**Y**^**a**^	**Y**	**Dec. 2016**	**Common cold sign, sore throat, chill earache, headache, enlarged lymph node, amaurosis, sore eye**	**Not treated**	**Only spring**	**N**	**N**	**562**	**1:160**	**≥400**	**92**
**10**	**77/F**	**Preauricular**	**N**	**N**	**Nov. 2016**	**Sore throat, enlarged lymph node (removed by surgery)**	**Not treated**	**Only spring**	**Y**	**N**	**576**	**1:160**	**195**	**120**
**11**	**31/F**	**Pharyngeal**	**N**	**N**	**Feb. 2018**	**Earache toothache enlarged lymph node**	**Co-amoxiclav/ 2 month**	**Only Tap water**	**Y**	**Y**	**115**	**1:80**	**32**	**≤3**

*Y* Yes, *N* No, *Pos.* Positive, *Neg.* Negative, *O.* Obtained, *NT* Not Taken, *ND* Not determined, *LNS* Lymph node sample, *WBS* Whole blood sample, *SS* Serum sample, ^a^Drained pus 1

### Rodent sampling

Rodent sampling was carried out around the affected village and its springs. Trapping was conducted using traditional live-catch traps with baits (dates, cucumber and cheese puffs). Rodents were identified using morphological traits as identification keys [21, 22]. Serum and spleen samples from rodents were transferred at 4 °C to the national Reference Laboratory for Plague, Tularemia and Q fever, at the Pasteur Institute of Iran laboratory.

### Culturing of rodent samples

Samples of rodents’ spleen were homogenized with sterile saline. To investigate the presence of *F. tularensis*, 50 μl of homogenized spleen samples were inoculated to sheep blood agar and Cysteine Heart Agar with 9% chocolatized sheep blood (CHAB) made selective by adding Cycloheximide, Vancomycin, Trimethoprim, Sulfamethoxazole and Colistin [23]. Agar plates were incubated in 5% CO_2_-enriched atmosphere at 37 °C for a week. Bacterial growth was monitored daily.

### Culturing water samples

From each water source, six samples (2.5 l per sample) were collected. Water samples were transferred to the laboratory at 4–8 °C within 48 h. Each sample was filtered on two 0.22-μm filters using a vacuum pump concentration system (Millipore, Burlington, Massachusetts, United States). The first filter was used for culture and divided into two parts. One part was directly inoculated to CHAB medium with antibiotics. The second part was first inoculated to the enrichment broth medium T [24] for 48 h, and then cultured on CHAB medium. The second filter of each sample was used for direct DNA extraction from the re-suspended filtrate in sterile PBS buffer. After 72 h incubation of the plates, DNA extraction was performed for all bacteria cultivated. All the extracted DNAs from different samples were tested by Real-Time PCR assays.

### Genome extraction and real-time PCR testing

DNA was extracted from each rodent spleen sample using the High Pure PCR template preparation kit (Roche). Real-time PCR testing for detection of *Francisella* spp. targeted the *ISFtu2* elements (Table 2) [25], using the Rotor-Gene device (model 6000, Corbett Life Science) and previously described protocols [19]. *ISFtu2 *positive samples were tested using *fopA* real-time PCR to confirm *F. tularensis* infection (Table 2) [26]. The NCTC 10857 Strain of *F. tularensis* was used as a positive control.

**Table 2:** Primers and probes used for detection of *F. tularensis*

Gene target	Primer and probe	Sequence (5′ to 3′)	Amplicon size (bp)
IS*Ftu2*	ISFtu2-F	TTGGTAGATCAGTTGGTAGGATAACC	97
	ISFtu2-R	TGAGTTTTATCCTCTGACAACAATATTTC	
	ISFtu2-Probe	FAM-AAAATCCATGCTATGACTGATGCTTTAGGTAATCCA-TAMRA	
*fopA*	fopA-F	AACAATGGCACCTAGTAATATTTCTGG	87
	fopA-R	CCACCAAAGAACCATGTTAAACC	
	fopA- Probe	FAM-TGGCAGAGCGGGTACTAACATGATTGGT-TAMRA	

### Standard tube agglutination procedure for detection of antibodies against *F. tularensis*

Standard tube agglutination was performed on all human and rodent serum samples according to the protocol of the manufacturer (Bioveta Inc., ivanovice, Czech Republic) to detect antibodies against *F. tularensis*. Antibody titers ≥80 were considered positive, and a titer at 40 was considered suspicious [27].

### ELISA

Furthermore, the presence of Anti-*F. tularensis* IgG and IgM antibodies in patients’ serum samples were tested using ELISA quantitative kits (Serion/ Virion, Germany). Anti-*F. tularensis* IgG and IgM antibodies titers were determined using a standard curve (as recommended by the manufacturer), and amounts of IgM and IgG were reported as U/ml. According to the kit’s protocol, values less than 10 U/ml were considered negative, titers between 10 and 15 U/ml borderline, and those higher than 15 U/ml positive.

## Results

All 11 examined patients’ sera had anti-*F. tularensis* antibodies of the IgM type, with very high titers (above 195 U/ml) in nine patients. Ten patients also had high IgG titers, whereas IgG was not detected in one patient (case No. 11). The tularemia tube agglutination test was positive in ten patients, and an ambiguous titer (1:40) was found for a patient (Table 1).

Real-time PCR tests revealed that one out of 5 water samples belonging to one of the springs of the village was positive for *ISFtu2* elements and *fopA* gene.

One *Microtus socialis* out of nine captured rodents from around the main well of the village was positive for *ISFtu2* elements and *fopA* gene. The negative rodent samples belonged to *Microtus socialis* (2)*, Microtus mystacinus* (1)*, Dryomys nitedula* (1)*, Apodemus witherbyi* (2)*, Arvicola persicus* (1) and *Meriones persicus* (1).

No positive result was obtained by culture or serological testing for any of the rodent samples. Direct culture attempts using filtered water that was injected into laboratory animals did not yield *F. tularensis*. It was not possible to isolate *F. tularensis* from human lymph node and blood samples, nor from rodent spleen and water samples.

After confirmation of the tularemia outbreak, patients were treated with doxycycline, 200 mg daily in two oral doses, for at least 15 days. Dredging and chlorination of the main water storage tank of the village were done. Villagers were encouraged to use tap water and received training to boil the spring water before drinking. Training programs for villagers about tularemia and its transmission modes were provided by distributing educational brochures, holding face-to-face training courses and sharing related educational materials in the virtual spaces in which the villagers were active. Training courses about clinical symptoms and treatment of tularemia were given to physicians and health care workers within 1 month after outbreak confirmation. Two years after the outbreak verification and the interventions, all the patients were cured, and no new cases of the disease were reported.

## Discussion

Here we present the first report of a probably tularemia outbreak in Iran. The extent of the outbreak and its probable source were determined by combining human, animal and environmental data.

More than 200 species of small mammals are known reservoirs of *F. tularensis* [1]. In a previous study performed in 2013 in Kurdistan province (western Iran), the seropositive rodents belonged to *Microtus, Dryomys* and *Meriones* [18]. In the present study, one rodent (*Microtus socialis*), out of nine collected and tested, was PCR-positive for *F. tularensis*. It is likely that rodents, like in many tularemia endemic areas, have an important role in the maintenance and transmission of this disease in Iran. Population growth in rodents is often associated with the occurrence of epizootics of tularemia, with massive contamination of the aquatic environment with *F. tularensis* [28]. The remarkably high density of rodents reported in the agricultural lands of the village in the summer of 2016 could have triggered the tularemia outbreak in this area.

We could not detect *F. tularensis* in all collected water samples, except for one of the spring water sources, but it has previously been reported that the time, type and location of water sampling play a major role in the ability to detect this pathogen during a tularemia outbreak [4].

In this outbreak, nine out of 11 evaluated patients had a disease compatible with typical oropharyngeal tularemia. This form of the disease is usually transmitted through drinking contaminated water of eating food washed with contaminated water. Tularemia is an endemic disease in Turkey that is the northwestern neighbor of Iran and located near this outbreak region [29–31]. From 1988 to 2009, 1300 cases of tularemia were reported in different regions of Turkey [6], almost all cases corresponding to the oropharyngeal form of the disease [6, 12]. Two patients involved in this outbreak suffered from swelling and redness of eyes, but without local lymphadenopathy as usually reported in the oculoglandular form of tularemia. This clinical form is transmitted through splashing or washing eyes with *F. tularensis*-contaminated water, or touching eyes with contaminated fingers. It is usually rare, accounting for less than 1% of human tularemia cases. Ocular forms of tularemia have been reported in Turkish patients [6]. Occasionally, both the oropharyngeal ad oculoglandular forms of tularemia may be combined. Interestingly, nine out of 11 patients diagnosed with tularemia had been suffering from this disease for more than 1.5 years and underwent various therapeutic and surgical treatments. However, due to the lack of diagnosis and proper treatment, they were not completely cured. Delay in diagnosis and treatment of tularemia cases in regions where tularemia is rarely reported is common, as the clinical signs of this disease are similar to those of a wide range of other infectious diseases [1].

In the present study, we used serological methods to diagnose tularemia cases. Serological tests represent the most commonly used method for tularemia diagnosis [32]. Specific antibodies against *F. tularensis* are usually detectable 10 to 20 days following disease onset [1, 32]. In a compatible clinical and epidemiological context, a single serum sample with a high antibody titer against *F. tularensis* antigen is considered diagnostic for tularemia. We could not isolate *F. tularensis* from human, rodent or aquatic samples. The sampling of patients was performed very late during the course of their disease, when bacteria had already been eliminated from the body by the immune system. In addition, most patients had already received several courses of antibiotic therapy. In patients with tularemia, the isolation of *F. tularensis* is usually successful only in the acute stage of infection, before a significant immune response has been mounted and appropriate antibiotic therapy has been administered. Culture of water and rodent samples and injection of these samples to laboratory animals did not allow isolation of *F. tularensis*. Previous studies also reported that the isolation rates of *F. tularensis* from environmental samples are very low [33]. Despite the fact that *F. tularensis* were not isolated in this outbreak investigation, the epidemiological and clinical evidence indicates that the outbreak was likely caused by *F. tularensis* subsp. *holarctica*. Until now, only this subspecies has caused tularemia in Europe and Asia. Human infections with type B strains commonly occurred from contaminated aquatic sources, including springs, wetlands, ponds, and rivers, and also from aquatic and semi-aquatic rodents [4].

Likely sources of human infections during this outbreak were the communal village tap water system and spring waters from the local environment. There are several pieces of evidence supporting this hypothesis: 1) the oropharyngeal form of tularemia was predominant, 2) all involved patients had drunk from the same tap or spring water (two drank only tap water, five cases only spring water, and four cases both tap and spring water), 3) there was a lack of proper chlorination of tap water during a long period before the outbreak, 4) an infected rodent was collected from the proximity of the main well resource of the village, and 5) improving prevention measures, including proper chlorination of the tap water supply allowed control of the tularemia epidemic.

In conclusion, lack of awareness of tularemia clinical manifestations in the general population and in health care workers, as well as the usual mild severity of type B tularemia cases likely explain that this disease is poorly diagnosed in Iran. In addition, specific tularemia diagnostic tests have been lacking for years in this country and have become available again only recently.

## Conclusion

The investigations of this outbreak and other recent serosurvey studies confirm that tularemia is an endemic disease in Iran. It is suggested that training programs be made available to physicians and healthcare personnel in all the country to raise awareness about the sources and transmission routes of *F. tularensis* and clinical signs of tularemia. Further studies are also needed to better evaluate the role of water contamination, animal reservoirs and potential vectors of tularemia in Iran. Investigating the levels of *F. tularensis* contamination of surface waters and infection rates in humans, livestock and wildlife animals in different regions would better clarify the endemic situation of tularemia in Iran.

## Data Availability

The datasets used during the current study are available from the corresponding author on reasonable request.

## Abbreviations

PCR: Polymerase chain reaction
ELISA: Enzyme-linked Immunosorbent Assay
*F. tularensis*: *Francisella tularensis*
